# miR-509-3-5P inhibits the invasion and lymphatic metastasis by targeting PODXL and serves as a novel prognostic indicator for gastric cancer

**DOI:** 10.18632/oncotarget.16802

**Published:** 2017-04-04

**Authors:** Jing Zhang, Zhonglin Zhu, Jinxin Sheng, Zhilong Yu, Bin Yao, Kejian Huang, Lisheng Zhou, Zhengjun Qiu, Chen Huang

**Affiliations:** ^1^ Department of General Surgery, Shanghai General Hospital, School of Medicine, Shanghai Jiao Tong University, Shanghai 200080, China; ^2^ Department of General Surgery, Haimen People's Hospital, Haimen 226100, Jiangsu province, China

**Keywords:** miR-509-3-5p, PODXL, invasion, lymphatic metastasis, gastric cancer

## Abstract

**Background:**

Our study aimed to investigate the clinicopathological feature and prognostic role of miR-509-3-5P in gastric cancer, to determine the invasive and metastatic role of miR-509-3-5P *in vitro* and *in vivo* and to explore the molecular mechanism between miR-509-3-5P and PODXL.

**Results:**

Strikingly lower miR-509-3-5P expression was detected in gastric cancer tissues with advanced tumor stage, poor differentiation and advanced pT stage, and was regarded as an independent prognostic role for poor prognosis. MiR-509-3-5P expression was markedly down-regulated in gastric cancer cell lines and tissues comparing with normal gastric cell and adjacent normal tissues, respectively. Decreased expression of miR-509-3-5P promoted the colony, migration and invasion abilities of gastric cancer cells *in vitro* as well as tumorigenesis and lymph node metastasis *in vivo*. Based on the luciferase assay and tissue microarray, PODXL was regarded as a target gene of miR-509-3-5P.

**Materials and Methods:**

The expression of miR-509-3-5P in gastric cancer patients and its clinicopathological relationships as well as prognostic role was studied employing tissue microarray; qRT-PCR was applied to explore miR-509-3-5P expression in gastric cancer cell lines and samples. Moreover, public database was used to analyze the expression of miR-509-3-5P and PODXL. Functional and molecular mechanism experiments were performed *in vitro* and *in vivo*.

**Conclusions:**

Overexpression of miR-509-3-5P inhibits the invasion and metastasis of gastric cancer *in vitro* and *in vivo*, functioning as a tumor suppressor, by targeting PODXL. More importantly, miR-509-3-5P was downregulated in gastric cancer tissues and may serve as a novel prognostic indicator for gastric cancer.

## INTRODUCTION

Gastric cancer (GC), one of the malignant cancer in digestive system, accounts for about 7% of all cancer cases and 9% of all cancer-related death worldwide [1]. However, in China, the morbidity and mortality of GC are both ranked the second with more than 90 000 new cases and 70 000 mortalities annually [2]. Despite of striking improvements in surgery and subsequent radiotherapy and chemotherapy, the prognosis of patient with advanced stage is still poor, especially those having lymph node metastasis. Hence, illustration of the molecular mechanism regulating tumor aggression and lymph node metastasis is robustly significant.

MicroRNAs (miRNAs), a group of small noncoding RNA gene products about 19~25 nucleotides in length, are clarified to be involved in various tumor development and progression [3, 4]. Generally, miRNAs bind to messenger RNAs’ (mRNAs) 3′ untranslated region (3′ UTR), which result in the degradation or translated inhibition of mRNA [5]. Emerging evidence suggests that altered expressions of miRNAs are implicated in various tumor biological process, such as cell growth, proliferation, angiogenesis, migration, invasion and so forth, via regulating targeted genes, [6–8]. Kong et al. demonstrated that miR-511, acting as tumor suppressor, could inhibited the abilities of proliferation of GC via targeting TRIM24 [9]. Additionally, it was found that miR-208-3P, overexpressed in GC, could suppress apoptosis of GC cells by downregulating PDCD4 and then promote the development of GC *in vivo* [10]. The critical roles of miRNAs involved in tumor metastasis, which was a pivotal step for poor prognosis, was further supported by various studies [11, 12]. Recently, a self-assembled cell microarray was applied to detect the specific miRNAs mediating GC metastasis, and the final results revealed that miR-451, downregulated in human GC specimens, could suppress the metastasis of GC via targeting ERK2 [13]. Moreover, it was found by Tsai et al. that decreased expressions of miR-26b in fresh GC tissues were not only associated with advanced clinicopathological stage, but also correlated with distant metastasis. And the decreased expression of miR-26b was correlated with poor 5-year survival. Further *in vitro* and *in vivo* experiments uncovered that enforced expression of miR-26b could result in the suppression of invasion and metastasis by targeting KPNA2/c-jun pathway [14]. It is well known that lymph node metastasis, a critical step for distant metastasis, plays an irreplaceable role in the progression of GC. And a few studies manifested that the development of lymphatic metastasis was affected by miRNAs [15, 16]. However, little was known about the lymphatic metastasis role of miRNAs in GC. In our present study, we will explore the role and mechanism of miR-509-3-5P, screened by miRNA microarray, in GC.

In our current study, miRNA microarray was firstly used to detect the potential differential expressed miRNAs in N0 (with no lymph node metastasis) and N3 (with more than 7 lymph node metastasis) group of GC. Numerous miRNAs were found to be reduced or increased in N3 group, which might be related to lymph node metastasis. Thereafter, the level of potential differential expressed miRNAs in GC tissues and paired adjacent normal tissues were verified. And it was found that miR-509-3-5P was downregulated in GC cancer tissues, which was correlated with advanced pN stage. *In vitro* and *in vivo* assays were further applied to manifest the tumor suppressor role of miR-509-3-5P in GC. Moreover, we confirmed that PODXL was one of the targeted genes of miR-509-3-5P via molecular biology. Our findings provided that miR-509-3-5P, one of the tumor suppressor, could prevent tumor invasion and lymph node metastasis via targeting PODXL in GC, and act as a novel prognostic indicator for GC.

## RESULTS

### miR-509-3-5P was decreased in GC and correlated with clinical features

**Figure 1:**
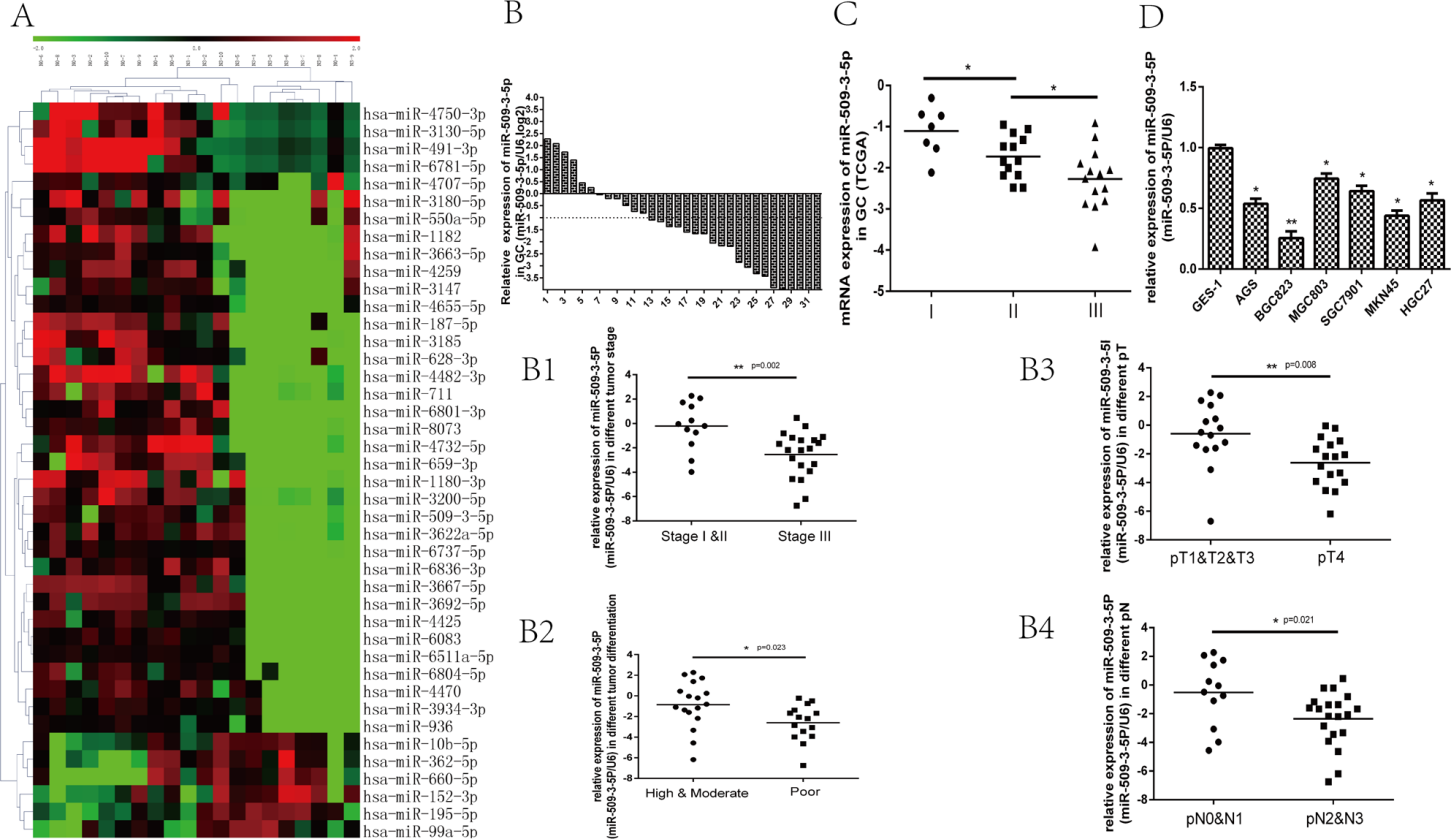
The potential differential expressed miRNAs between N0 and N3 group, and the expression of miR-509-3-5P in fresh GC tissues, the Cancer Genome Atlas (TCGA) database and GC cell lines (**A**) The potential differential expressed miRNAs (increase or decrease at least 2-fold, *p* < 0.05) between N0 and N3 group screened by miRNA microarray. (**B**) the expression of miR-509-3-5P in 32 GC tissues and adjacent normal tissues was determined by qRT-PCR. MiR-509-3-5P expression was observed to be decreased in 26 (81.25%) GC tissues. The logarithmic scale 2^−ΔΔCt^ was applied for the analysis. B1-B4. The correlations between miR-509-3-5P and tumor stage, differentiation, pT stage, pN stage in 32 fresh GC tissues. (**C**) The TCGA data showed that miR-509-3-5P level was decreased gradually in stage I, stage II, stage III. (**D**) miR-509-3-5P expression in six different GC cell lines in contrast with normal human gastric epithelial cells-1 ( *p < 0.05, **p < 0.01).

**Figure 2:**
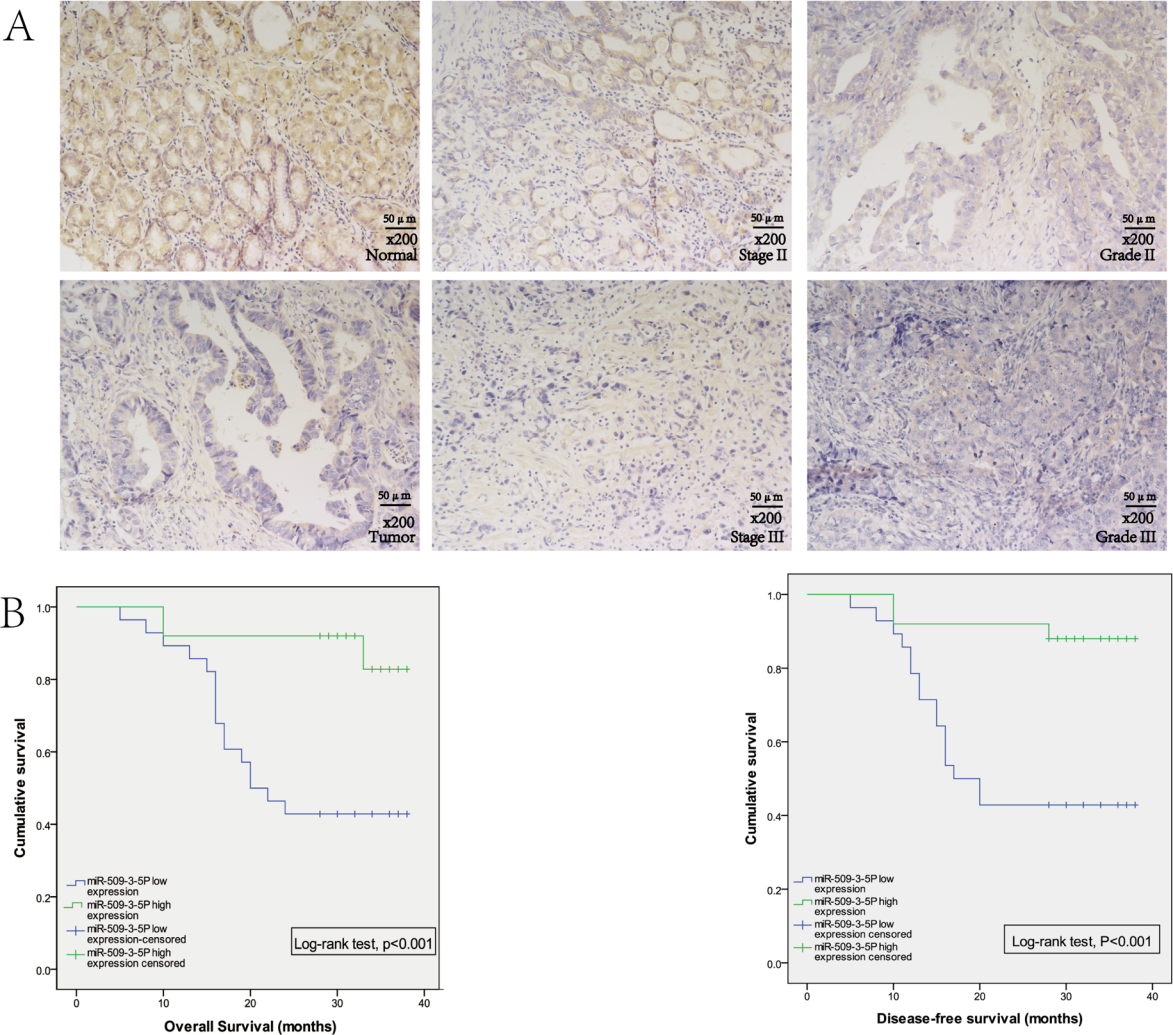
The expression of miR-509-3-5P in GC tissues microarray (**A**) *In situ hybridization* was used to detect the expression of miR-509-3-5P in GC tissues microarray. Representative images of miR-509-3-5P expression of normal gastric mucosa and GC tissues were shown in left column. The middle column revealed the expression of miR-509-3-5P in tumor stage II and stage III. And the right column showed the expression of miR-509-3-5P in Grade II and Grade III. (**B**) The relationships between miR-509-3-5P expression and overall survival (OS), disease-free survival (DFS) in GC tissues. Patients with high expression of miR-509-3-5P showed a significant longer OS (*p* < 0.001) and DFS (*p* < 0.001). Kaplan-Meier method was used to evaluate the OS and DFS, log-rank test was applied to evaluate the difference.

First of all, miRNA microarray was applied to detect the potential differential expressed miRNAs in 10 tissues of GC with N0 (with no lymph node metastasis) and 10 tissues of GC with N3 (with more than 7 lymph node metastasis), we found that miR-509-3-5P, miR-1180-3P, miR-936 and so forth were downregulated in N3 group (Figure 1A), which might be associated with lymph node metastasis. Thereafter, we verified the level of potential differential expressed miRNAs in 32 GC tissues and paired adjacent normal tissues. And we then found that miR-509-3-5P was downregulated in fresh tissues of GC (Figure 1B), and the decreased miR-509-3-5P expression was not only correlated with advanced clinicopathologic stage (UICC stage), but associated with poor differentiation, tumor stage(pT stage), lymph node metastasis (pN stage).

(Figure 14). Further public data from TCGA revealed that reduced miR-509-3-5P expression was closely associated with advanced clinicopathologic stage (Figure 1C). In order to further elucidate the expression of miR-509-3-5P in paraffin-embedded specimens of GC, ISH was applied in tumor tissues and adjacent normal tissues. Interestingly, the expression of miR-509-3-5P detected in tumor tissues was significant lower in comparison with those in adjacent normal tissues (Figure 2A). Moreover, the observations of ISH study supplied unequivocal evidence that lower expression of miR-509-3-5P was significantly correlated with advanced clinicopathologic stage, pT stage, pN stage and poor differentiation (Figure 2A and Table 1). Thus, we put forward an assumption that low expression of miR-509-3-5P, negatively associated with pathological parameters, may contribute to the aggressiveness of GC and function as a predictor for poor prognosis.

**Table 1:** correlation between miR-509-3-5P expression and clinicopathological parameters in GC (*n* = 54)

Parameters	category	No.	MiR-509-3-5P expression		*p*
			low	high	
Age					0.055128
	< 65	26	17	9	
	≥ 65	28	11	17	
Gender					0.439419
	Male	38	21	17	
	Female	16	7	9	
T stage^1^					0.022088*
	T2	5	0	5	
	T3	21	10	11	
	T4	28	18	10	
N stage	N0	11	3	8	0.027214*
	N1	12	4	8	
	N2	13	7	6	
	N3	18	14	4	
UICC stage^2^	II	17	4	13	0.007740**
	III	37	24	13	
Nerve invasion	Yes	29	17	12	0.283639
	No	25	11	14	
Vessel invasion	Yes	29	14	15	0.571098
	No	25	14	11	
Differentiation^3^	High	3	0	3	0.000101***
	Moderate	19	4	15	
	Low	32	24	8	
Tumor size	≤ 3	23	12	11	0.967456
	> 3	31	16	15	

^1,2^There is no T1 stage tumor or M1 patients in our GC tissues microarray.

^3^Low differentiation includes poor differentiation, mucinous adenocarcinoma, and signet ring cell carcinoma.

*p < 0.05, **p < 0.01,***p < 0.001.

### Lower expression of miR-509-3-5P may serve as an independent biomarker for poor prognosis in GC

To better determine the independent predictive value of miR-509-3-5P expression level, univariate and multivariate analysis for DFS and OS were carried out via Cox proportional hazards model (Table 2). Univariate analysis showed that pN stage, UICC stage and miR-509-3-5P expression were associated with prognosis of GC, and multivariate analysis further suggested that lower level of miR-509-3-5P (HR = 0.232, 95% CI:0.064–0.842, *p* = 0.026) was closely related to poor prognosis. Kaplan-Meier survival analysis and the log-rank test for OS and DFS survival curves were used to reveal the relationship between miR-509-3-5P expression and patients’ survival. GC Patients with higher expression of miR-509-3-5P had a significantly better DFS and OS rate than that with lower expression of miR-509-3-5P (Figure 2B), implying that miR-509-3-5P might serve as an independent prognostic indicator for GC patients.

**Table 2:** univariate and multivariate analysis for GC overall survival (OS) and disease-free survival (DFS)

Parameters	No.	Overall survival				Disease-free survival			
		unvariate analysis		Multivariate analysis		unvariate analysis		Multivariate analysis	
		HR (95% CI)	P	HR (95% CI)	P	HR (95% CI)	P	HR(95% CI)	P
Age			0.732				0.826		
< 65	26	1.171 (0.475–2.886)				1.106 (0.449–2.723)			
≥ 65	28								
Gender			0.695			0.819 (0.295–2.275)	0.702		
Male	38	0.815 (0.293–2.265)							
Female	16								
T stage			0.107				0.101		
T2	5	1.924 (0.868–4.264)				1.957 (0.878–4.365)			
T3	21								
T4	28								
N stage			0.005		0.145		0.005		0.137
N0	11	2.056 (1.240–3.409)		1.723 (0.829–3.580)		2.052 (1.242–3.390)		1.742 (0.839–3.616)	
N1	12								
N2	13								
N3	18								
UICC stage			0.034		0.999		0.035		0.977
II III	17 37			(0.120–8.331)					
Nerve invasion			0.165				0.164		
Yes No	29 25	0.503				0.503			
		(0.191–1.325)				(0.191–1.324)			
Vessel invasion			0.776				0.770		
Yes	29	0.876 (0.352–2.179)				0.873 (0.351–2.170)			
No	25								
Differentiation			0.162				0.154		
High	3	1.922 (0.769–4.804)				1.951 (0.778–4.891)			
Moderate poor	19 32								
Tumor size			0.699				0.758		
≤ 3	23	0.837 (0.340–2.061)				0.868 (0.353–2.136)			
> 3	31								
MiR-509-3-5P			0.004		0.026		0.004		0.021
Low	28	0.165 (0.048–0.569)		0.232 (0.064–0.842)		0.160 (0.046–0.551)		0.221 (0.061–0.799)	
high	26								

HR: hazard ratio; CI: confidence interval.

*p < 0.05 indicate that the 95% CI of HR was not included.

### MiR-509-3-5P was downregulated in GC cell lines and inhibited cell migration and invasion *in vitro*

**Figure 3:**
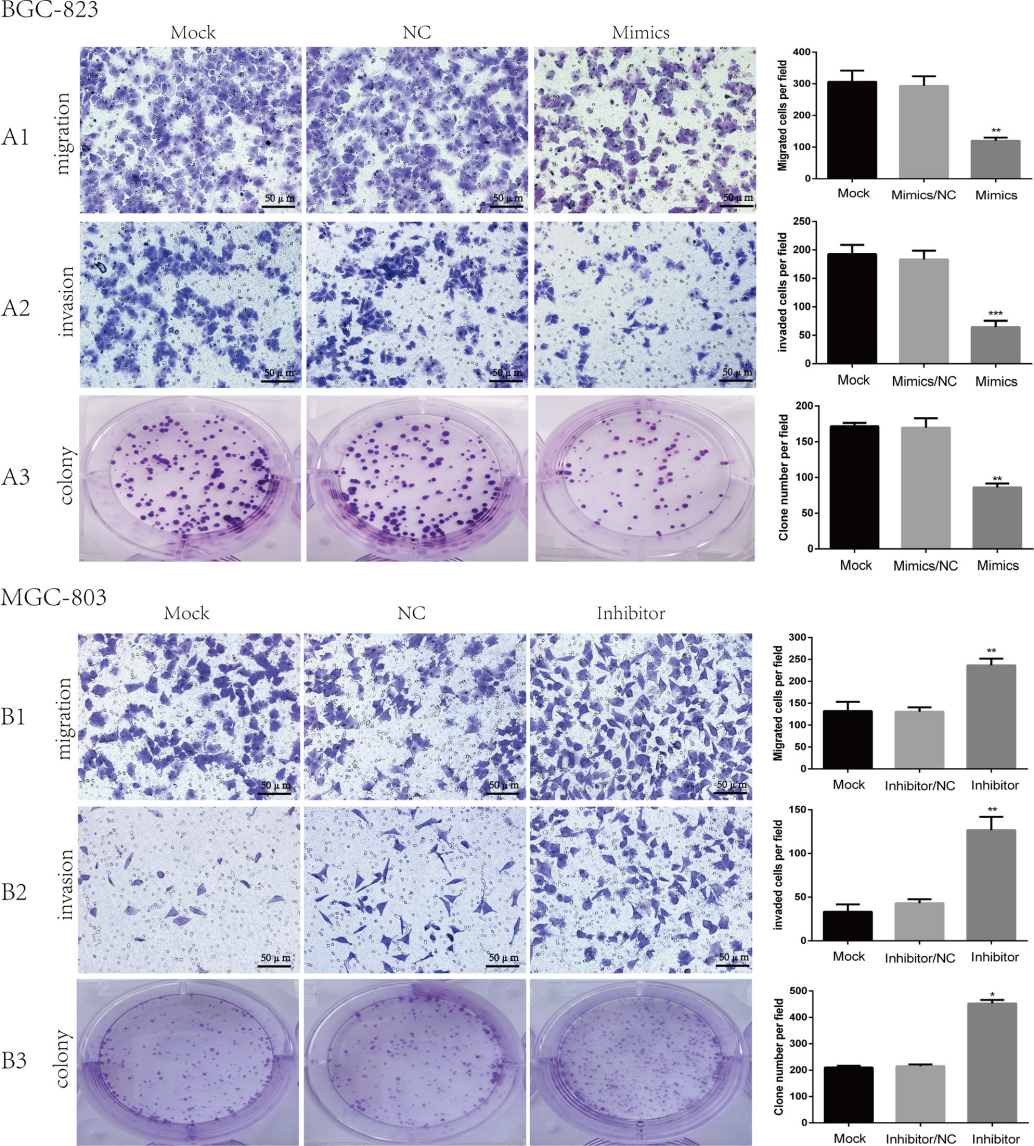
The role of miR-509-3-5P in the migration, invasion and clonogenicity of BGC823, MGC803 (**A1**–**A3**) Representative images of BGC823 migration, invasion and colony in mock, miR-509-3-5P/negative control (NC), and miR-509-3-5P/mimics group. Overexpression of miR-509-3-5P by mimics can inhibit the migration, invasion and colony abilities in BGC823. (**B1**–**B3**) Representative images of MGC803 migration, invasion and colony in mock, miR-509-3-5P/negative control (NC), and miR-509-3-5P/inhibitor group. Downregulation of miR-509-3-5P by inhibitor can enhance the migration, invasion and colony abilities in MGC803. Mean ± SEM was shown for these data (*p < 0.05, **p < 0.01, ***p < 0.001).

In order to examine exact miR-509-3-5P expression in GC cell lines, qRT-PCR was implemented. It was confirmed that expression of miR-509-3-5P was relatively lower in GC cell lines in contrast with that tested in GES-1 (Figure 1D). And then BGC-823, showing the lowest miR-509-3-5P expression, and MGC-803, having the highest miR-509-3-5P expression, were selected for further dispose to investigate biological processes (migration, invasion and clonogenicity), which were vital for tumorigenesis and metastasis. The BGC823 cells were transfected with miR-509-3-5P mimics and negative control (NC), MGC803 cells were transfected with miR-509-3-5P inhibitors and NC, respectively. It was demonstrated that the migration and invasion abilities were reduced in BGC823 with miR-509-3-5P overexpression as compared with the cells of mock and NC group (Figure 3A1 and 3A2). However, inhibition of miR-509-3-5P in MGC-803 increased their migration and invasion abilities (Figure 3B1 and 3B2). Similarly, the colony number of BGC823 transfected with mimics was lower than that in mock and NC group (Figure 3A3). Conversely, MGC803 with reduced miR-509-3-5P expression showed higher clonogenicity in comparison with that in mock and NC group (Figure 3B3). Above assays revealed that miR-509-3-5P inhibited migration, invasion and clonogenicity of GC cells *in vitro*.

### Podocalyxin-like 1 (PODXL) is a target gene of miR-509-3-5P

**Figure 4:**
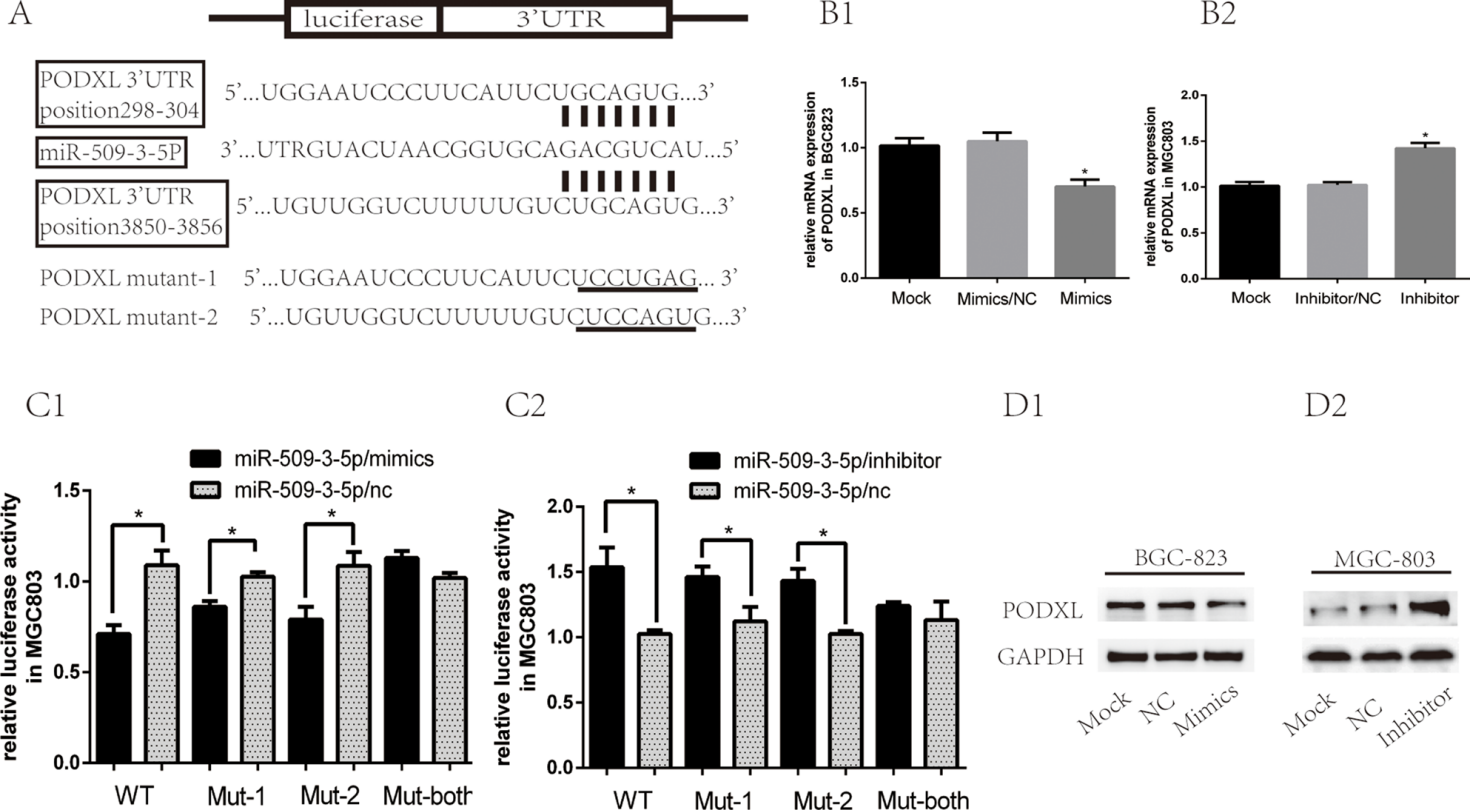
MiR-509-3-5P suppressed expression of PODXL via directly binding to its 3′UTR (**A**) There were two potential binding sites of miR-509-3-5P at the 3′-UTR of PODXL mRNA based on the bioinformatic analysis. 3′-UTR of PODXL mRNA containing wild type, mutant-1, mutant-2, mutant-both of was cloned into dual luciferase plasmid. (**B1**–**B2**) The mRNA expression of PODXL in BGC823 transfected with none, miR-509-3-5P/NC, miR-509-3-5P mimics and MGC803 transfected with none, miR-509-3-5P/NC, miR-509-3-5P/inhibitor. (**C**) Wild type (WT) or mutant-1(mut-1) or mutant-2 or mutant-both PODXL 3′-UTR was transfected into MGC803 with miR-509-3-5P/NC or miR-509-3-5P mimics or miR-509-3-5P/inhibitor, respectively. Firefly and Renilla luciferase signals were performed for luciferase activity after 36 h transfection. (**D1**–**D2**) The expression of PODXL in BGC823, MGC803 transfected with none, miR-509-3-5P/NC and miR-509-3-5P mimics or miR-509-3-5P/inhibitor, was measured by Western blot after 48 h transfection. Mean ± SEM was shown for these data.

To better investigate the mechanism of miR-509-3-5P in GC aggressiveness, bioinformatic analysis was used to search its candidate target genes. PODXL, one of the oncogene, was selected from the predicted targets intersected from Targetscan,microRNA.org and pita (Figure 4A). Firstly, we found that the mRNA and protein of PODXL in BGC823 transfected with mimics was reduced as compared with control group (Figure 4B1 and 4D1). When transfected with inhibitor in MGC803, the mRNA and protein of PODXL was elevated (Figure 4B2 and 4D2). To further confirm the effect of miR-509-3-5P on the 3′ UTR of PODXL mRNA, we then cloned the 3′UTR of PODXL(wt 3′UTR) or mutant sequence (mut 3′UTR) into a luciferase vector, which also contained the internal control luciferase gene—Renilla. After Luc-PODXL-wt plasmid and miR-509-3-5P/mimics were co-transfected into MGC803 and 293T cells, the luciferase activity was decreased significantly as compared with control group (Figure 4C1 and Supplementary 1A1). On the contrary, Luc-PODXL-wt plasmid co-transfected with miR-509-3-5P/inhibitor in MGC-803 and 293T revealed opposite effects (Figure 4C2 and Supplementary 1A2). Based on the prediction of 2 potential binding sites in the 3′UTR of PODXL of miR-509-3-5P, we constructed 3 mutant plasmids: luc-PODXL-mut-1 (potential first binding site mutation), luc-PODXL-mut-2 (potential second binding site mutation) and luc-PODXL-mut–both (potential both two binding sites mutation). Interestingly, both luc-PODXL-mut-1 and luc-PODXL-mut-2 attenuated miR-509-3-5P/mimics or miR-509-3-5P/inhibitors-mediated luciferase activity to certain degree (Figure 1, 2, 3, 42 and S1.A1-A2). However, transfection with PODXL-mut-both plasmid and miR-509-3-5P/mimics or miR-509-3-5P /inhibitors showed no difference in luciferase activity in contrast with control group (Figure 1, 2, 3, 42 and Supplementary 1A1–A2). Our results validated that miR-509-3-5P could directly bind to both predicated binding sites in 3′UTR of PODXL .

**Figure 5:**
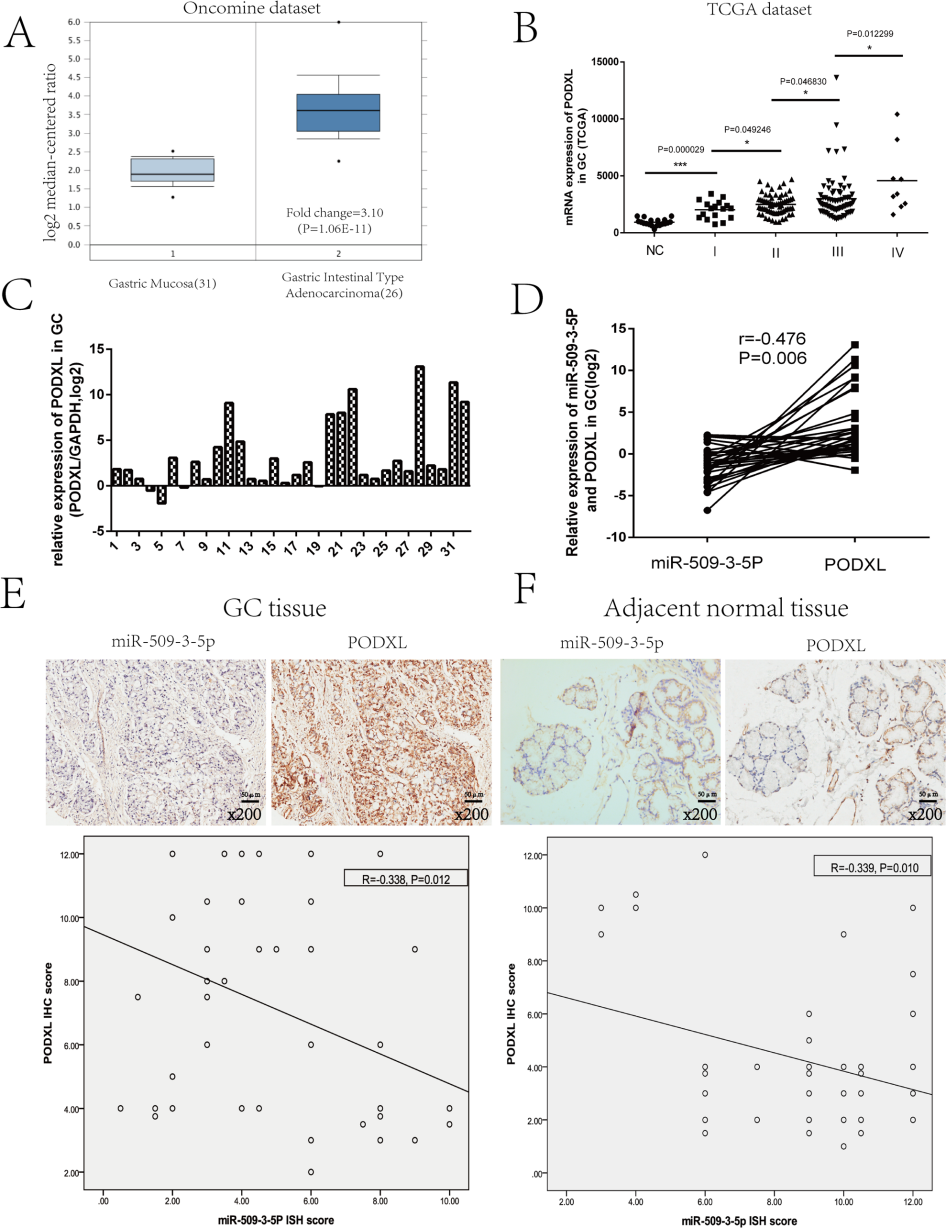
Analysis of PODXL expression in GC based on the Oncomine and TCGA database, and the correlation between miR-509-3-5P and PODXL GC (**A**) Exhibition of PODXL mRNA expression in GC tissues and normal tissues from Oncomine database (DErrico, 2009). (**B**) TCGA dataset showed that PODXL level of GC was increased in advanced tumor stage as compared with normal tissues. (**C**) The mRNA expression of PODXL was detected by quantitative real-time PCR(qRT-PCR) in 32 fresh GC tissues as compared to adjacent normal tissues. The logarithmic scale 2^−ΔΔCt^ was applied for the analysis. (**D**) Representative image of the correlation between miR-509-3-5P and PODXL mRNA expression in 32 GC tissues (Pearson's correlation analysis, *r* = −0.476, *p* = 0.006). (**E**–**F**) The relationship between miR-509-3-5P and PODXL in GC tissues microarray was determined by Pearson's correlation. Negative correlations were observed in GC tissues (*r* = −0.338, *p* = 0.012) and adjacent normal tissues (*r* = −0.339, *p* = 0.010).

More importantly, the qRT-PCR results and IHC score revealed that the correlations between miR-509-3-5P and PODXL were negative in fresh GC and paraffin-embedded GC tissues (Figure 5D, 5E and 5F), which further indicated that miR-509-3-5P could negatively regulate PODXL expression via directly binding to its 3′UTR.

### PODXL performs as a tumor promoter

Emerging evidence showed that PODXL, normally expressed on vascular endothelial cells, hemangioblasts, podocytes and so on [17], had been found to be overexpressed in several cancers, such as pancreatic cancer, hepatocellular, breast cancer, esophageal cancer and gastric cancer and behaved as a oncogene [18–20]. Public data (Oncomine and TCGA) showed that expression of PODXL was higher in GC tissues as compared with GC normal tissues (Figure 5A). Moreover, elevated expression of PODXL was associated with advanced UICC stage (Figure 5B). And our study further confirmed that PODXL was overexpressed in GC tissues (Figure 5C). However, the functions of PODXL in GC cells are not yet well clarified. To better explore its specific role in GC cells, BGC823 and MGC803 cells were chosen for overexpression and blockage of PODXL, respectively, confirmed by Western blotting (Supplementary Figures 2, 3). The abilities of migration, invasion and colony formation were significantly promoted in BGC823 cells with PODXL overexpressed as compared with mock and control group (Supplementary Figure 2). Conversely, blockage of PODXL in MGC803 cells resulted in the decrease in migration, invasion and colony formation (Supplementary Figure 3). Taken together, PODXL, as a tumor promoter, was involved in the invasion and metastasis of GC.

**Figure 6:**
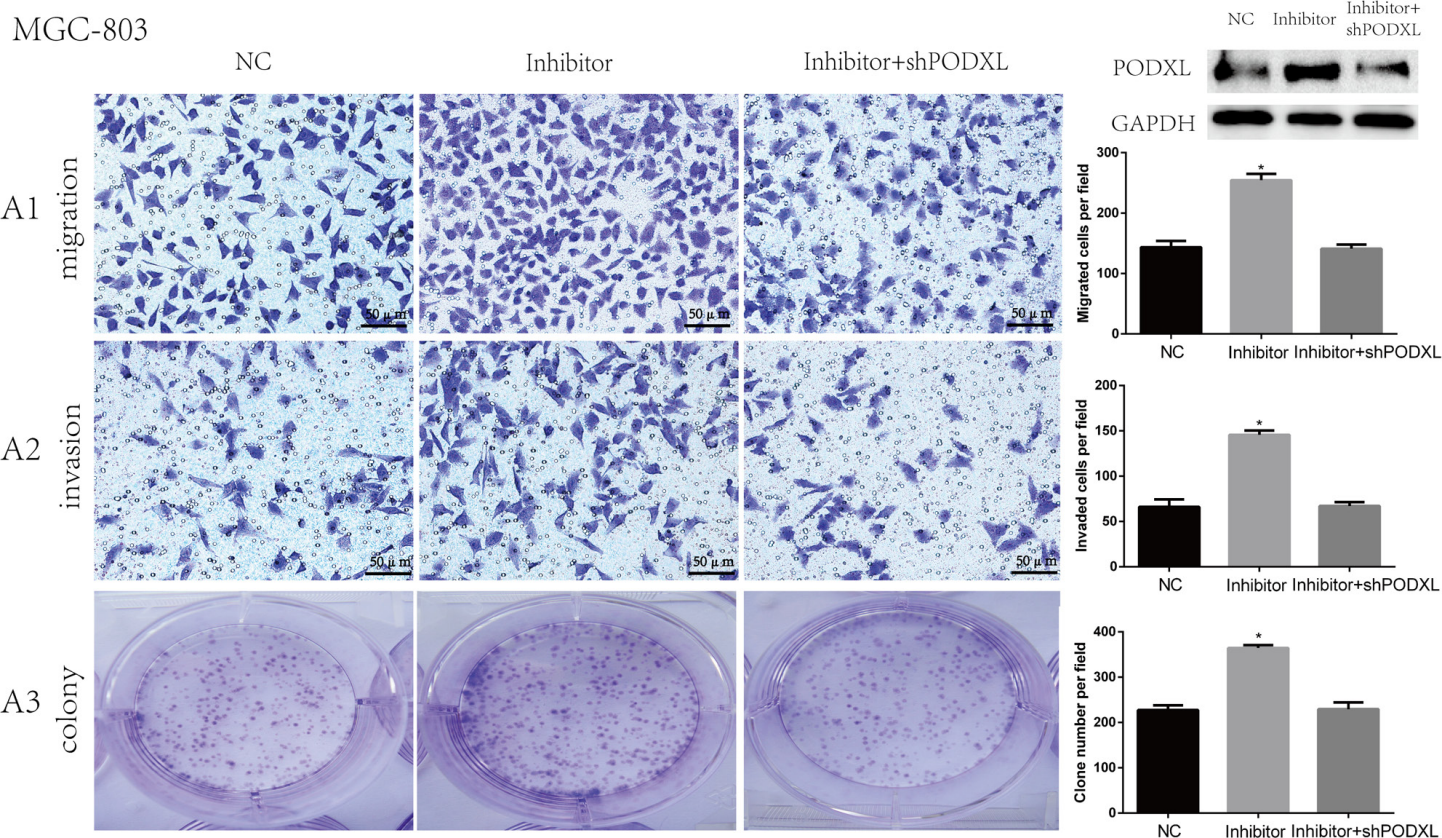
Knockdown of PODXL attenuated migration, invasion and colony formation mediated by miR-509-3-5P/inhibitor A1-A3. Representative images of MGC803 migration, invasion and colony in control plasmid (NC), miR-509-3-5P/inhibitor and miR-509-3-5P/ inhibitor+shPODXL group. Knockdown of PODXL attenuated migration, invasion and colony formation mediated by miR-509-3-5P/inhibitor. Mean ± SEM was shown for these data (*p < 0.05).

To further determine the role of miR-509-3-5P and PODXL on cell migration, invasion and colony, MGC-803 cells were co-transfected with miR-509-3-5P/inhibitor+shPODXL, miR-509-3-5P/inhibitor or control plasmid. As shown in Figure 6, blockade of miR-509-3-5P leaded to significant increase in cell migration, invasion and colony formation as compared with that transfecting with miR-509-3-5P/inhibitor+shPODXL. Those data indicated that PODXL contributed greatly in miR-509-3-5P induced cell biological process.

### miR-509-3-5P inhibits tumorigenicity and lymph node metastasis via repressing PODXL *in vivo*

**Figure 7:**
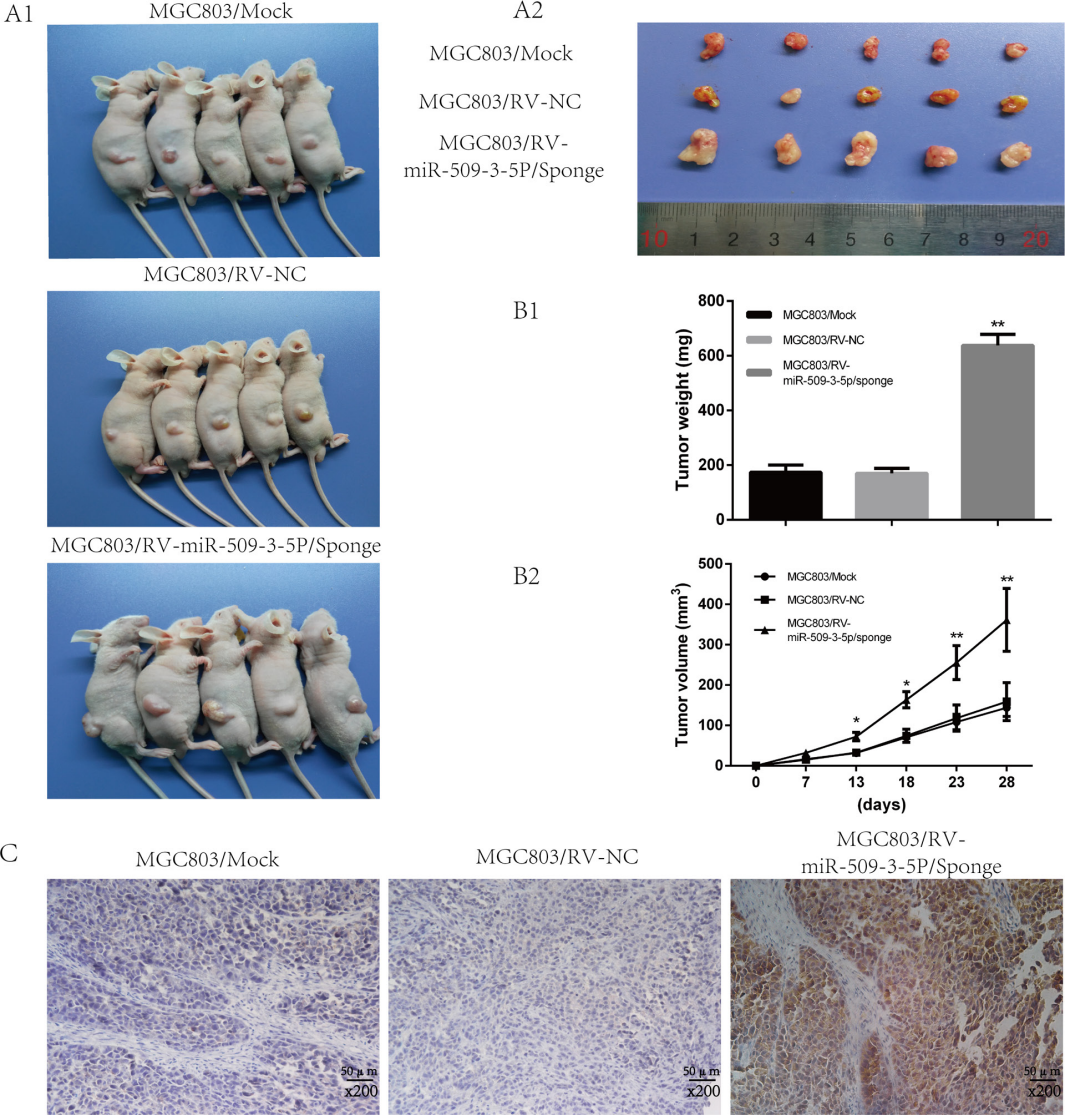
Sponged miR-09-3-5P expression promoted tumorigenicity *in vivo* (**A1**) Image of tumors of MGC803/mock, MGC803/RV-negative control (NC) and MGC803/RV-miR-509-3-5P/sponge group. (**A2**) The representative image of tumors excised from the mice. The tumors of MGC803/RV-miR-509-3-5P/sponge group were larger than other two groups. (**B1**) The representative graph was on behalf of tumor weight. (**B2**) The graph represented for tumor volume. (**C**) Representative image of PODXL expression in three different tumors obtained from nude mice by IHC. Obviously, the staining of PODXL in the tumor with less miR-509-3-5P was stronger than others.

In view of the functions of miR-509-3-5P *in vitro*, further investigation *in vivo* was carried out to examine the tumorigenicity and lymphatic metastasis. Stable cell line retrovirus-mediated MGC803/ miR-509-3-5P/sponge and MGC803/miR-NC were constructed and cultured as described. As illustrated in Supplementary Figure 1C2, the miR-509-3-5P expression in MGC803/ miR-509-3-5P/sponge accounted for a quarter of MGC803/miR-NC group or MGC803/mock group. After injecting subcutaneously into nude mice, the tumor of different groups were monitored, and the average tumor volumes of MGC-803 with miR-509-3-5P sponged were significantly larger compared with that in mock and NC group (Figure 7A1, 7A2). And the tumor volume and weight of MGC803 with miR-509-3-5P sponged developed rapidly than that in the mock and NC group (Figure 7B1 and 7B2). IHC results revealed that the tumors from MGC803 with miR-509-3-5P sponged exhibited higher expression of PODXL (Figure 7C). To sum up, these data further revealed that the rapid development of tumor might be partially contributed by PODXL overexpression caused by the inhibition of miR-509-3-5P. More importantly, the correlation between miR-509-3-5P and PODXL was opposite *in vivo*.

**Figure 8:**
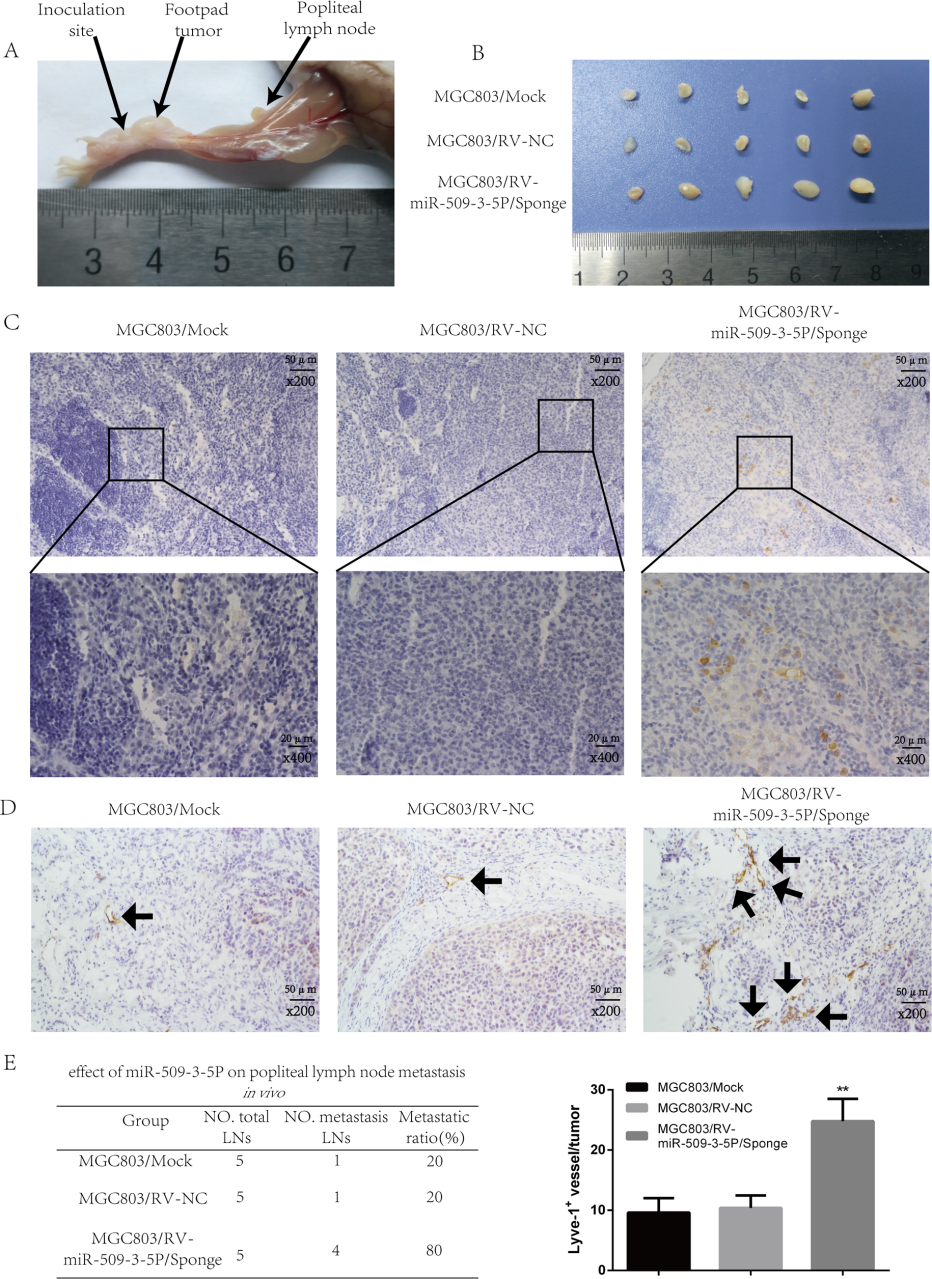
Sponged miR-09-3-5P expression promoted lymph node metastasis *in vivo* (**A**) The popliteal lymph node metastasis model was built by injecting the foot pad of nude mice with MGC803/mock, MGC803/RV-negative control (NC) and MGC803/RV-miR-509-3-5P/sponge cells. (**B**) Representative photograph of lymph nodes from nude mice dissected 4 weeks later after inoculation in foot pad. (**C**) Representative image of popliteal lymph nodes stained with anti-E-cadherin antibody. The upper panel was shown in 200×, the lower panel was shown in 400×. (**D**) The level of MLD marked by Lyve-1 in MGC803/RV-miR-509-3-5P/sponge group was higher than the rest two groups. (**E**) The left table showed the metastatic ratio of all dissected popliteal lymph nodes of nude mice. The right graph represented for the level of microlymphatic vessel density (MLD) in the tumor of three groups (*p < 0.05, **p < 0.01).

In order to further discover the effect of miR-509-3-5P on lymphatic metastasis *in vivo*, a popliteal lymphatic metastasis model was employed (Figure 8A). The above mentioned cells were injected into the foot-pads of nude mice separately. After 4-week feed, the lymph nodes and foot-pad tumors were collected and analyzed. The volumes of lymph nodes formed from MGC803/miR-509-3-5P/sponge had larger volumes compared with mock and control group (Figure 8B). And then the anti-human E-cadherin antibody was used to label MGC-803 cells in all lymph nodes (Figure 8C) [21], and we found that the lymph nodes in MGC803/miR-509-3-5P/sponge group had a high ratio of metastasis (Figure 8E). As shown in Figure 8D, the tumor formed by MGC803/ miR-509-3-5P/sponge developed more level of microlymphatic vessel density (MLD), marked by Lyve-1 positive microvessel, as compared with mock and control group. Taken together, the above results further showed that miR-509-3-5P could inhibit tumorigenesis and lymphatic metastasis in GC *in vivo*.

## DISCUSSION

As we all know that the morbidity of GC in East Asian is extremely higher, especially in China, South Korea and Japan. However, owing to the restraint of public consciousness and economy, the rate of early GC detection in China is much lower in contrast with Japan and South Korea. Moreover, despite of rapid improvements in surgery and subsequent treatment, the prognosis of GC patient with advanced stage is still poor, which contributes strikingly to the leading cause of cancer-associated death [2]. Because of tumor recurrence and metastasis, specially lymph node metastasis, it is well acknowledged that the 5-year survival rate of advanced GC is less than 20% [22]. Under this situation, figuring out the underlying molecular mechanisms that lead to the aggression and lymphatic metastasis may offer a different eyesight on the treatment of GC.

Recently, mounting evidence has revealed the significant roles of miRNAs implicated in normal and pathologic process of cells, and the molecular mechanisms between miRNAs and their target mRNAs are well elucidated [23]. Particularly, miRNAs, involved in various cancers, have been manifested to be associated with tumor aggression and metastasis [24, 25]. For instance, Tang et al. [26] found that expression of miR-200b and miR-200c was decreased in GC tissues, and the decreased expression of miR-200b and miR-200c in paraffin-embedded GC specimens was correlated with advanced T stage, clinical stage and lymph node metastasis, suggesting that miR-200b and miR-200c might occupy an irreplaceable role in GC progression. Additionally, it was demonstrated by Zheng et al. that miR-409-3P, downregulated in GC, was associated with the development and progression of GC. Further *in vitro* experiments revealed that overexpression of miR-409-3P in GC cell lines could suppress the migration and invasion and *in vivo* experiments showed that enforced expression of miR-409-3P could inhibited peritoneal dissemination and distal pulmonary metastasis [27]. Taken together, the above study elucidated that miRNAs was implicated in the development and progression of GC. However, little was known about the role of miRNAs in lymph node metastasis of GC. In our current study, potential differential expressed miRNAs between GC pN0 and pN3 group was firstly detected using miRNA microarray, and we found that miR-509-3-5P, miR-1180-3P, miR-936 and so forth were downregulated in pN3 group, which might be tightly associated with lymphatic metastasis. The detected miRNAs were then examined in fresh GC tissues and paired normal tissues, and we discovered that miR-509-3-5P expression was decreased in GC tissues, and the reduced miR-509-3-5P expression was correlated with advanced pN stage and other clinicopathologic parameters, consistent with TCGA data and ISH result analysis. Moreover, miR-509-3-5P was closely associated with prognosis and the low level of miR-509-3-5P may serve as an independent biomarker for poor prognosis in GC. All these outcomes further underlined that miR-509-3-5P expression was closely related with tumor invasion and metastasis, particularly lymphatic metastasis, acting as a tumor suppressor in GC. Recently, we confirmed that the role of miR-509-3-5P in GC was similar to its suppressor role in lung cancer according to the limited literature [28].

Interestingly, diverse *in vivo* and *in vitro* experiments revealed that miRNAs also had effects on tumor growth, proliferation, migration, invasion, metastasis and so forth [29, 30]. Kim et al. demonstrated that overexpression of miR-10b could promote the proliferation, migration and invasion of breast cancer cell *in vitro* [31]. Kogo et al manifested that overexpression of miR-218 could suppress the migration and invasion of cervical cancer *in vitro* [15]. Currently, our *in vitro* experiments revealed that enforced expression of miR-509-3-5P inhibited the migration, invasion and colony formation abilities, whereas blockage of miR-509-3-5P resulted in the opposite effects. In terms of subcutaneous xenograft, blockade of miR-509-3-5P could promote the tumor growth, more importantly, it could also facilitate the lymphatic metastasis *in vivo*. And all the *in vivo* and *in vitro* experiment indicated that miR-509-3-5P could act as a tumor suppressor in GC.

It is well known that miRNAs bind to targeted mRNAs via perfect or imperfect base pairing, contributing to the degradation of targeted mRNAs or suppression of their translation [32]. Normally, there are potential numerous targeted mRNAs targeted by a specific miRNA. For example, it was demonstrated detailedly that miR-10b could regulate transcription factor TBX5 in breast cancer [31], E-cadherin in laryngeal carcinoma [33], HOXD10 in GC [34] etc. MiR-509-3-5P, less discussed nowadays, could lead to anti-proliferative effect in human lung cancer A549 cells by targeting PLK1 [28]. However, the role of miR-509-3-5P in tumor invasion and metastasis was rarely reported, not to mention its role in GC. In our present study, we found that miR-509-3-5P was downregulated in GC tissues, and lower expression of miR-509-3-5P was

correlated with advanced clinicopathologic parameters and poor prognosis in GC. Nevertheless, our study uncovered that PODXL was regarded as a target gene of miR-509-3-5p in GC from different perspectives. First and foremost, PODXL was regarded as a predicted targets from bioinformatic analysis. Moreover, the mRNA and protein levels of PODXL in GC cell lines was reduced when transfected with miR-509-3-5P mimics, and vice versa. Additionally, the luciferase activity of PODXL luciferase reporter was decreased when elevating miR-509-3-5P expression. Opposite effects were acquired when co-transfected with miR-509-3-5P inhibitors. While mutating the two binding sites in the 3′UTR of PODXL, no difference was observed. Furthermore, functional overlap, including migration, invasion and colony assays, could be observed between miR-509-3-5P and PODXL. Finally, the results of clinical specimens and subcutaneous tumor xenograft samples further disclosed a negative correlation between miR-509-3-5P and PODXL. The above mentioned outcomes verified that PODXL ought to be a targeted gene of miR-509-3-5P, indicating that miR-509-3-5P might be involved in GC invasion and metastasis via negatively regulating PODXL.

PODXL, not only an anti-adhesive transmembrane glycoprotein but also a CD 34 ortholog, normally expressed on vascular endothelial cells, hemangioblasts, podocytes and so on [17]. Mounting evidence had demonstrated that PODXL, overexpressed in pancreatic cancer, hepatocellular, breast cancer, esophageal cancer, colorectal cancer and so forth, was considered as a tumor promoter. Moreover, some studies verified that overexpression of PODXL was correlated strongly with advanced clinicopathological features (poor differentiation, advanced tumor stage etc.) and poor prognosis [18–20, 35–37]. In our current study, we confirmed that PODXL was overexpressed in GC tissues based on the results of qRT-PCR, IHC and the database of TCGA. In terms of the role of PODXL *in vitro*, we found that the migration and invasion abilities were closely associated with PODXL expression. Blockade of PODXL could inhibit GC cell migration and invasion, enforced expression of PODXL did the opposite effect, which were consistent with Taniuchis’ results in pancreatic cancer [38]. It was reported by Catherine et al. that elevated expression of PODXL was closely related with lymphatic invasion in breast cancer [39]. While in our research, we found that downregulation of miR-509-3-5P, which relatively expressed high levels of PODXL, could promote lymphatic metastasis in nude mice. Taken together, we concluded that decreased expression of miR-509-3-5P could promote aggression and lymphatic metastasis of GC via negatively regulating PODXL.

Apparently, our study indicated that elevated expression of miR-509-3-5P inhibited PODXL expression via binding directly to its 3′UTR. However, it is undeniable that some others binding sites are existed in 3′UTR of PODXL, For instance, miR-5100, miR-199b-5P are treated as negative regulators of PODXL in pancreatic cancer [35] and acute myeloid leukemia [40]. Thus, we could not exclude the possibility that these miRNAs would be implicated in the regulation of PODXL expression in the progression of GC. In addition, little is known about the role of other potential target genes of miR-509-3-5P in the development of GC, which may be involved in signaling pathways. Taken together, further studies are needed to uncover the regulation of miR-509-3-5P in the GC aggression.

To sum up, our results clearly revealed that miR-509-3-5P, an important antionco-miRNA associated with lymphatic metastasis, were decreased in GC tissues and cell lines. Ectopic expression of miR-509-3-5P in GC cell lines inhibited the colony, motility and invasion abilities via negatively targeting PODXL. Moreover, ISH results uncovered that low expression of miR-509-3-5P could be a significant biomarker for advanced clinicopathological features, lymphatic metastasis and poor prognosis in GC. Besides, the decreased expression of miR-509-3-5P led to larger xenograft tumor and more lymphatic metastasis in nude mice. Collectively, our study not only offers miR-509-3-5P as a novel prognostic indicator for GC, but also uncovers an aberrant miR-509-3-5P-PODXL signaling pathway, which may provide a promising eyesight for designing novel agent to control GC aggression and lymph node metastasis.

## MATERIALS AND METHODS

### Patients and specimens

32 pairs of fresh gastric cancer tissues and adjacent normal tissues were collected after radical surgical resection in the Shanghai General Hospital. After resection, the tissues were transported in liquid nitrogen and stored at −80°C refrigerator for RNA and protein extraction. 57 paired gastric cancer and adjacent normal tissues, collected from 2013 to 2014, were paraffin-embedded for the tissue microarray (TMA) construction (The final TMA contained 54 GC tissues and 57 adjacent normal tissues). The patients enrolled in TMA construction were followed every 3 months for 2 years and then every 6 months for later 3 years, and finally annually. All patients mentioned above had never received radiotherapy and chemotherapy before surgery. Clinicopathological feature was diagnosed and confirmed by two pathologists based on the guidelines of the International Union against Cancer (UICC). Written informed consent for each patients was obtained before enrolling in the study. And the study protocol was approved by the Ethical Committee for Clinical Research of Shanghai General Hospital affiliated Shanghai Jiaotong University.

### Cell lines

The human gastric cancer cells, including AGS, BGC823, MGC803, SGC7901, MKN45, HGC27, and the normal human gastric epithelial cells-1 (GES-1) were obtained from the Type Culture Collection of the Chinese Academy of Science ( Shanghai, China). Cells mentioned were maintained in 1640/F12K medium with 1% penicillin-streptomycin and 10% fetal bovine serum (FBS) (Gibco, Carlsbad, CA). All the cells were fostered at 37°C in a humidified atmosphere filled with 5% CO_2_.

### The Oncomine and the cancer genome atlas (TCGA)

The mRNA expression of PODXL in GC tissues and normal mucosae was acquired from Oncomine (www.oncomine.org). The expression of miR-509-3-5P and PODXL in different tumor stage was downloaded from TCGA website (https://tcga-data.nci.nih.gov/tcga/). RNA-Seq analysis used from TCGA was that there were 7 stage I, 13 stage II, 14 stage III GC for miR-509-3-5P, 17 stage I, 68 stage II, 73 stage III, 9 stage IV GC and 19 adjacent normal tissues for PODXL

### RNA extraction and quantitative real-time PCR(qRT-PCR)

Based on the manufacturer's instructions, total RNA was extracted from gastric cancer cell lines, gastric cancer tissues and surrounding normal tissues using TRIzol reagent ( Invitrogen, Carlsbad, CA). Each RNA was transcribed into complementary DNA using PrimeScript RT reagent kit (Takara, Shiga, Japan). qRT-PCR was performed employing SYBR Premix Ex Taq^™^ (Tli RnaseH Plus; Takara) on a ViiA 7 fast real-time PCR system (Applied Biosystems, NY, USA). U6 and GAPDH were used as an internal control for miRNA and mRNA, respectively. And the ΔΔCt method was applied to evaluate relative expression levels. The specific primers used in research were as follows: PODXL sense: 5′ TCTTGCCACCAGGACACCTA- 3′, antisense: 5′-TACCTCTTCCCAGACCCAAT-3′; GAPDH sense: 5′-GGACCTGACCTGCCGTCTAG-3′, antisense: 5′ -GTAGCCCAGGATGCCCTTGA-3′. All of the experiments were carried out at least in triplicate.

### Transient transfection

The has-miR-509-3-5P mimics, has-miR-509-3-5P inhibitor and corresponding negative controls were synthesized from RiBoBo (Guangzhou, China). The short hairpin RNA (shRNA) specially targeting PODXL and pcDNA-PODXL plasmid were purchased form Genechem Co., Ltd. (Shanghai, China). shRNA-PODXL sense: TATCAGTGAGATCAATTTC, Antisense: GAAATTGATCTCACTGATA. Gastric cancer cells in logarithmic growth phase were seeds in 6-well plates with adequate numbers for the transfection. The oligonucleotides targeting different genes was performed employing Lipofectamine^™^ 2000 in terms of the manufacturer's instructions (Invitrogen).

### Cell migration and invasion assays

For these assays, the Corning transwell insert chambers were either covered with Matrigel (BD Biosciences, USA ) to detect the ability of invasion or nothing to detect the ability of migration. Specific GC cells were trypsinized, resuspended in serum-free medium and counted by counting plate in triplicate. In the upper chamber, about 4 ×10^4^ prepared cells were incubated, and the lower chamber was supplied with 600 uL medium containing 10% FBS. After 24 h incubation at 37°C in the incubator, the cells migrating and invading through membrane were fixed with 20% methanol, then dyed with crystal violet, and finally photographed and counted. All experiments were carried out independently in triplicate.

### Colony formation assay

In order to evaluate colony formation, a thousand transfected GC cells were cultured in 6-well plate with complete growth media for 2 weeks at most. Then cell colonies were fixed with methyl alcohol for 30 min, stained with crystal violet for another 30 min, then washed, imaged and counted. Experiments mentioned were carried out independently in triplicate.

### Protein extraction and western blotting

Total protein was separated from GC cell using Radio-Immunoprecipitation Assay (RIPA) with the protease inhibitor phenylmethanesulfonyl fluoride (Beyotime Biotechnology, Jiangsu, China), and then the concentration of protein was measured according to the manufacturer's instructions of BCA protein assay kit (Beyotime Biotechnology, Jiangsu, China). Equal protein (30 ug) was separated by 8% sodium dodecyl sulfate-polyacrylamide gel electrophoresis (SDS-PAGE), and isolated protein was soon transferred onto PVDF membranes. The membranes with separated protein were thereafter blocked in 5% fat-free milk dissolving in TBST buffer at room temperature for 1.5 h. Next, the membranes were incubated with specific primary antibody, respectively, at 4°C overnight. After 1.5 h incubation of a secondary antibody at room temperature, the protein was visualized using ECL regent (Millipore, MA, USA). The following antibodies were applied: PODXL(1:100; Santa Cruz, CA, USA), GAPDH(1:1000; Cell Signaling Technology, MA, USA).

### Luciferase assays

For this assay, MGC803 and 293T cells were harvested and seeded in 24-well plate with approximately 1 × 10^5^ cells per well. After 24 h incubation, about 200 ng wild-type or mutant PODXL 3-‘UTR psiCHECK-2 plasmid purchased from HarO Life Biotechnology Co., Ltd.(Shanghai, China), was cotransfected with 60pmol miR-509-3-5P/mimics or negative control and miR-509-3-5P/inhibitor or negative control into MGC-803 and HEK-293T cells applying 2 ul Lipofectamine^™^ 2000 reagent (Invitrogen). After 36 h transfection, the cell lysates were harvested and then the luciferase activities were detected by Dual-Luciferase Reporter Assay System (Promega, USA). All experiments mentioned were carried out independently in triplicate.

### Retroviral transduction for stable cell lines

MiR-509-3-5P-knockdowned vector pLenR-GPH or control vector pLenR-GPH was cotransfected with packaging plasmid mix into 293T cells to produce recombinant lentivirus. Then GC cells were infected with the Lentivirus generated by 293T cells, which was harvested at 48 h. Thereafter, the infected GC cells were purified with puromycin (Invitrogen). The stable cell lines MGC803/RV-miR-509-3-5P/ sponge and MGC803/RV-miR-negative control (NC) were all constructed by Biolink Biotechnology (Shanghai, China).

### Nude mice xenograft models

For *in vivo* study, 4-week-old BALB/C nude mice purchased from Shanghai Research Center for Model Organisms were randomly divided into 3 group (*n* = 5). 1 × 10^7^ MGC803/mock, MGC803/RV-negative control (NC) and MGC803/RV-miR-509-3-5P/sponge cells were subcutaneously injected into a nude mouse. Then the tumor nodules in mice were measured weekly with a caliper. Tumor volume was calculated employing the formula: volume = 1/2 × width^2^ × length. All mice were sacrificed on day 28, then the xenograft tissues were excised, weighed and paraffin embedded. The 4.0 um sections were taken and detected by immunohistochemistry using primary antibodies: PODXL. All animals used were approved by the Institutional Animal Care and Use Committee of Shanghai General Hospital, and all efforts were performed to relieve animal suffering.

### Popliteal lymph node metastasis model

4-week-old BALB/C nude mice purchased from Shanghai Research Center for Model Organisms were randomly divided into 3 group (*n* = 5). 4 × 10^6^ MGC803/mock, MGC803/RV-negative control (NC) and MGC803/RV-miR-509-3-5P/sponge cells were injected into the left footpads of mice. 4 weeks later, popliteal lymph nodes and the primary tumors were excised and paraffin-embedded. The 4.0 um sections were taken and detected by immunohistochemistry using primary antibodies: Lyve-1, E-cadherin. All animals used were approved by the Institutional Animal Care and Use Committee of Shanghai General Hospital, and all efforts were performed to relieve animal suffering.

### Immunohistochemistry (IHC) and *in situ* hybridization (ISH)

GC tissues microarray for IHC were firstly dewaxed in xylene and rehydrated in the gradient ethanol (100%, 95%, 85%, 75%). Then the microarray was prepared for antigen retrieval by boiling in 10 mM sodium citrate buffer (PH 6.0) for 5 min. After blockage of endogenous peroxidase activity using 3% hydrogen peroxide for 10 min, the slides were incubated with primary antibody, including PODXL antibody (1:200, Santa cruz, CA, USA, Cambridge, UK), Lyve-1 (1:100, Abcam, Cambridge, UK), E-cadherin (1:200, Cell signaling Technology, Massachusetts, USA), at 4°C overnight. With incubation of secondary antibody for 30 min at room temperature, the sections were then dyed with diaminobenzidine and Mayer's hematoxylin in turn. Later, the microarray was dehydrated in the gradient ethanol (75%,85%,95%, 100%), and finally covered with coverslips.

After deparaffinization the microarray of GC for ISH was incubated with proteinase K at 37°C for 10 min. Thereafter, the slides were hybridized with hybridization mix at 57°C for 1 hour, then washed and incubated with hydrophobic barrier. Afterwards, the sections was blocked by blocking solution in a humidifying chamber for 15 min, and next incubated with the 10ng 3′-5′ DIG-labeled miR-509-3-5P probes overnight at 50°C. Washed twice stringently, the sections were covered with rabbit antibody aimed at digoxigenin and horse radish peroxidase on the basis of manufacturer's recommendation. After rinsed and dehydrated, the slides were mounted with coverslips.

The scores of GC samples were carried out by two independent pathologist blind to clinical data. The scores of the GC sections were critically depended on the proportion of staining area and intensity of staining. The proportion of staining area was labeled as 0 (0–10%), 1 (10–25%), 2 (26–50%), 3 (51–75%), 4 (76–100%). The intensity of staining cells were scored as 0 (negative staining), 1 (weak staining), 2 (moderate staining) or 3 (strong staining). The final score of each section was amounted to the multiplication of above two scores. According to the total score of each sample, tissues with score ≤ 4, and > 4 were treated as low and high expression.

### Statistical analysis

The significance of the data was calculated applying Student *t* test and Mann-Whitney *U* test for paired and unpaired continuous variables. The significances between miR-509-3-5P expression and clinicopathological features were determined by Fisher's exact test and the χ^2^ test. Survival curves were delineated employing Kaplan-Meier method, and the differences were calculated using log-rank test. The HR and significance between variables and overall survival (OS), disease-free survival (DFS) were carried out using Cox proportional hazards model. *P* value less than 0.05 was treated to be significant for all tests. All analyses mentioned were carried out applying SPSS 21 statistical software (SPSS Inc., USA).

## SUPPLEMENTARY MATERIALS FIGURES


