# Supplementary material for: miR-509-3-5P inhibits the invasion and lymphatic metastasis by targeting PODXL and serves as a novel prognostic indicator for gastric cancer
Source: Oncotarget. 2017 Apr 4;8(21):34867–83. doi: 10.18632/oncotarget.16802 (PMC5471018; doi:10.18632/oncotarget.16802)
Supplement: Supplementary file 1 [file oncotarget-08-34867-s001.pdf]

miR-509-3-5P inhibits the invasion and lymphatic metastasis by targeting PODXL and serves as a novel prognostic indicator for gastric cancer

Supplementary Materials

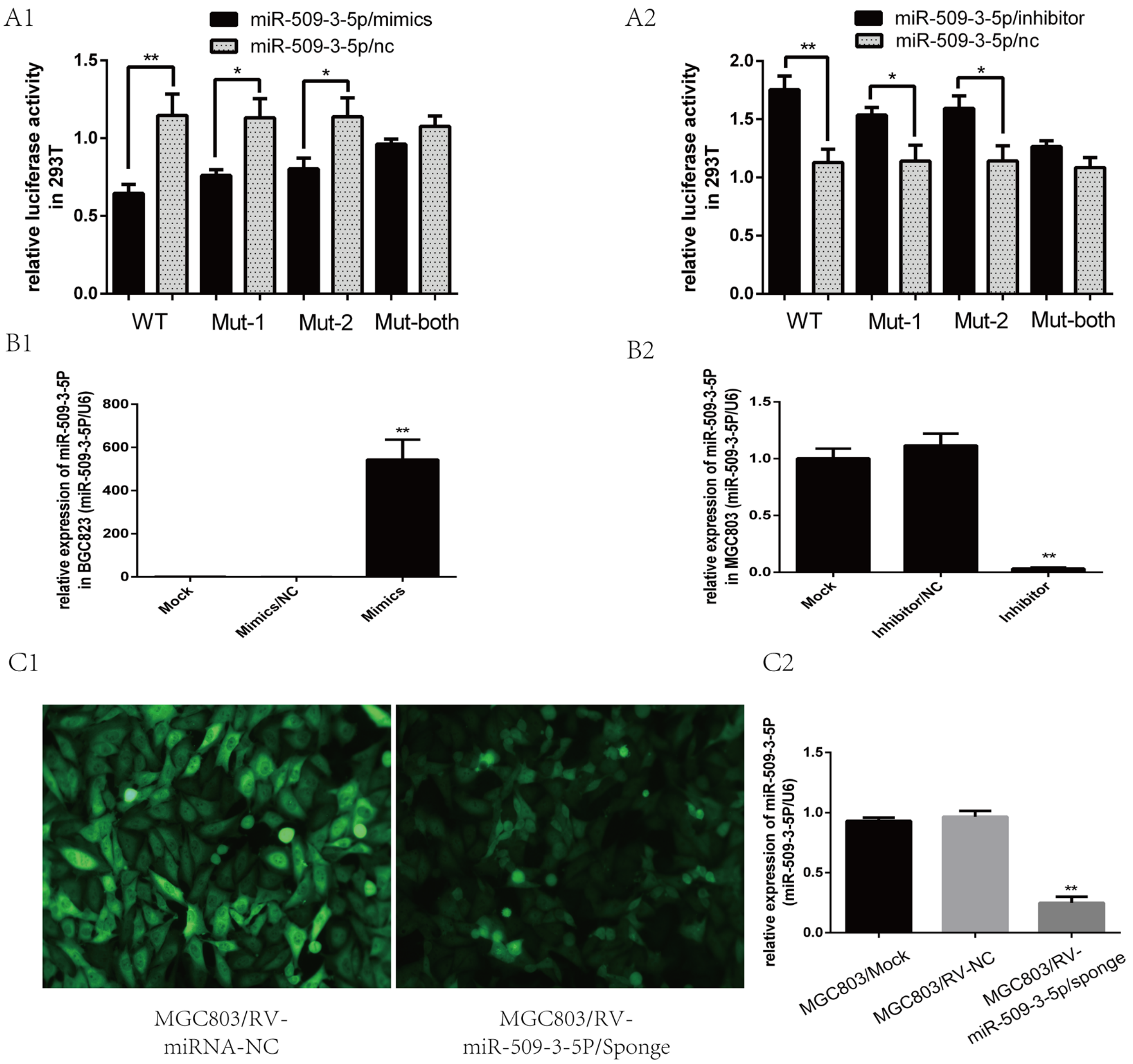

Supplementary Figure 1: The expression of miR-509-3-5P in BGC823 and MGC803. (**A**) Wild type (WT) or mutant-1(mut-1) or mutant-2 or mutant-both PODXL 3′-UTR was transfected into 293T with miR-509-3-5P/NC or miR-509-3-5P mimics or miR-509-3-5P/inhibitor, respectively. Firefly and Renilla luciferase signals were performed for luciferase activity after 36 h transfection. (**B1**–**B2**) The expression of miR-509-3-5P in BGC823 and MGC803 transfected with none, miR-509-3-5P/NC, miR-509-3-5P/mimics, miR-509-3-5P/inhibitor. qRT-PCR was used for the detection. (**C1**) Representative images of stable cell lines of MGC803 transfected with retrovirus miR-509-3-5P/NC, miR-509-3-5P/sponge. (**C2**) The expression of miR-509-3-5P in MGC803 transfected with miR-509-3-5P and miR-509-3-5P/sponge retrovirus, respectively. Mean ± SEM was shown for these data. (*$p < 0.05$, **$p < 0.01$).

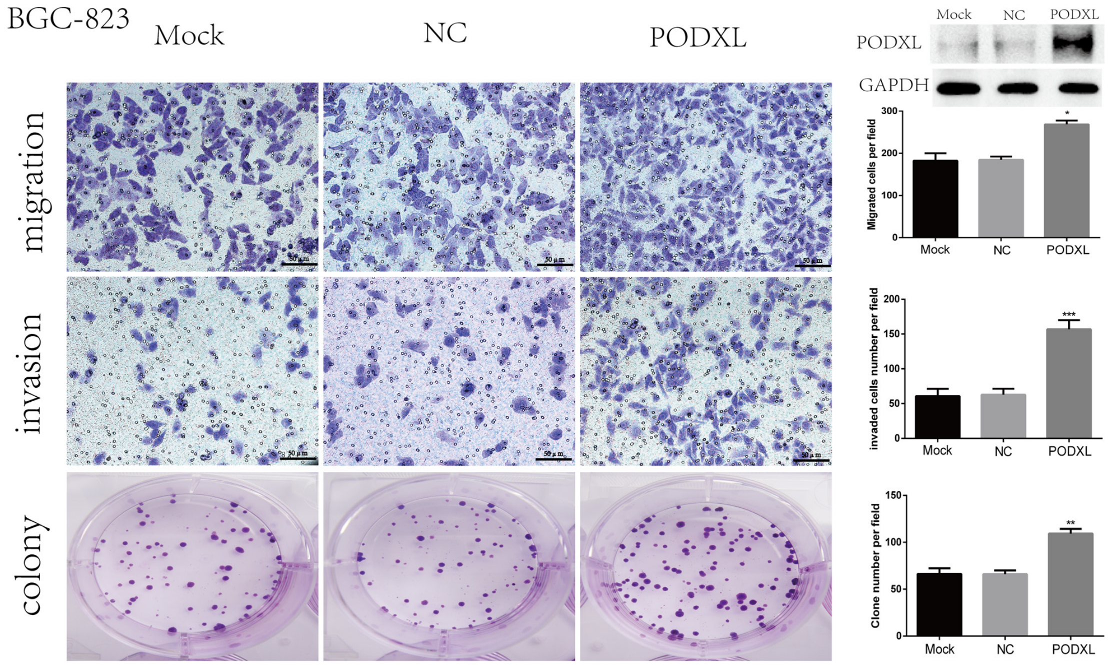

Supplementary Figure 2: The role of PODXL in the migration, invasion and clonogenicity of BGC823. Representative images of BGC823 migration, invasion and colony in mock, PODXL/negative control (NC), and PODXL group. Overexpression of PODXL can promote the migration, invasion and colony abilities in BGC823. Mean ± SEM was shown for these data. (**p* < 0.05, ***p* < 0.01, ****p* < 0.001).

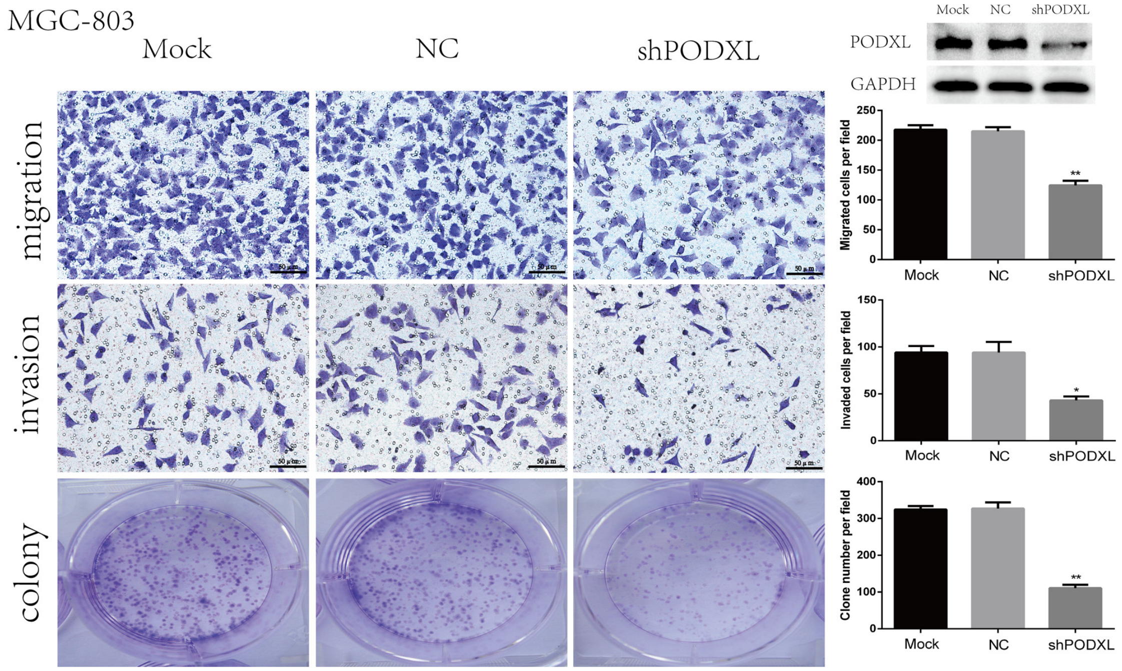

Supplementary Figure 3: The role of PODXL in the migration, invasion and clonogenicity of MGC803. Representative images of MGC803 migration, invasion and colony in mock, PODXL/negative control (NC), and PODXL group. Knockdown of PODXL can surppress the migration, invasion and colony abilities in MGCC803. Mean ± SEM was shown for these data. (**p* < 0.05, ***p* < 0.01).
